# CYP2C8 and antimalaria drug efficacy

**DOI:** 10.2217/14622416.8.2.187

**Published:** 2007-02-07

**Authors:** JP Gil, E Gil Berglund

**Affiliations:** 1 Karolinska Institute, Malaria Research Unit, Division of Infectious Diseases, Department of Medicine, Karolinska University Hospital, M9:02, KS 17176 Stockholm, Sweden. Tel.: +46 851 775 284; Fax: +46 851 776 740; jose.pedro.gil@ki.se; ^2^Medical Products Agency, Department of Preclinical and Clinical Evaluation 2, Uppsala, Sweden.; ^3^University of Algarve, Centre of Molecular and Structural Biomedicine, Portugal.

**Keywords:** allele, amodiaquine, artemisinine-based combination therapy, chloroquine, cytochrome P450 2C8, desethylamodiaquine, malaria, pharmacogenetics, pharmacokinetics

## Abstract

Malaria is a major infectious disease. In the last 10 years it has killed more than 20 million people, mainly small children in Africa. The highly efficacious artemisinine combination therapy is being launched globally, constituting the main hope for fighting the disease. Amodiaquine is a main partner in these combinations. Amodiaquine is almost entirely metabolized by the polymorphic cytochrome P450 (CYP) isoform 2C8 to the pharmacologically active desethylamodiaquine. The question remains whether the efficacy of amodiaquine is affected by the gene polymorphism. Genotype-inferred low metabolizers are found in 1–4% of African populations, which corresponds to millions of expected exposures to the drug. *In vivo* pharmacokinetic data on amodiaquine is limited. By combining it with published *in vitro* pharmacodynamic and drug metabolism information, we review and predict the possible relevance, or lack of, of CYP2C8 polymorphisms in the present and future efficacy of amodiaquine. Chloroquine and dapsone, both substrates of CYP2C8, are also discussed in the same context.

**Figure 1.**
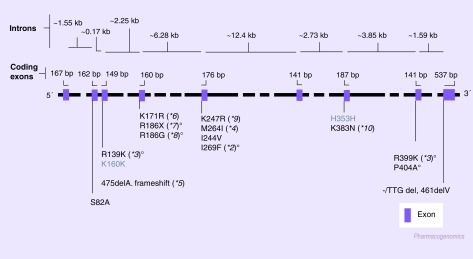
*CYP2C8* gene and known single nucleotide polymorphisms [101]. Only the single nucleotide polymorphisms present in the coding region of the gene are represented. ºAlleles coding for documented or predicted reduced enzyme activity; *2: Two-fold higher K_M_ for paclitaxel transformation [17]; *3: 15% of *1-associated turnover number [17]; *5: Expected to be an inactive gene [18]; *7: No *in vitro* detectable activity [21]; *8: *In vitro* enzyme activity 10% of the wild-type [21]; P404A: Reduced protein expression leading to decreased V_max_/K_M_ [32].

**Figure 2.**
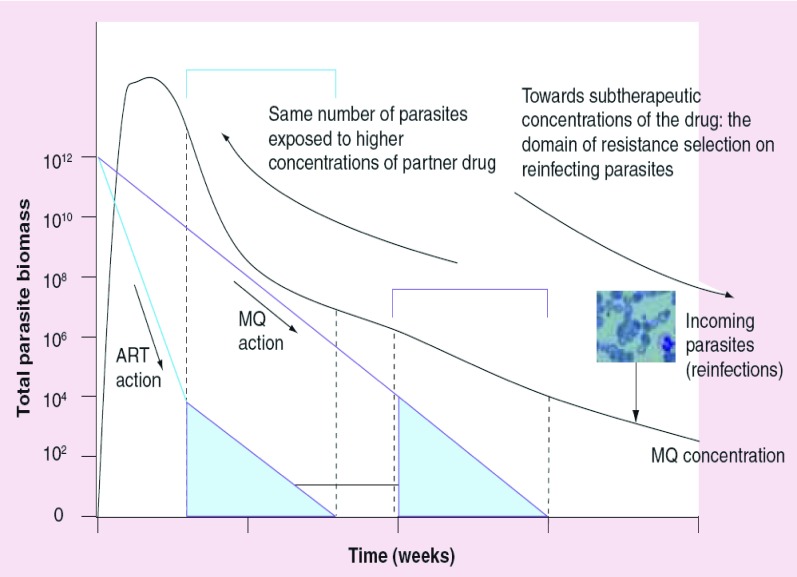
Artemisinine combination therapy. Artemisinine combination therapy is the main hope for the global control of malaria. The strategy is based on an elegant and simple concept, herein depicted from the example of the artesunate–mefloquine combination in current use in Thailand. The very fast parasite reduction rate (PRR) of artesunate (1/10,000) [78] rapidly reduces the parasite biomass, allowing the partner drug (mefloquine [MQ], with a substantial slower PRR, 1/100) to face a number of parasites several orders of magnitude smaller. No parasites remain when MQ serum concentrations reached subtherapeutic levels where selection of tolerants might ocour. The expected different mechanisms of action of these structurally different drugs, associated with their documented *in vitro* pharmacodynamic synergy, further contributes to protect the combination from resistance. Figure adapted from [4] and [22]. *P. falciparum* image courtesy of B Schmidt, Department of Medicine, Karolinska Institute, Sweden.

**Figure 3.**
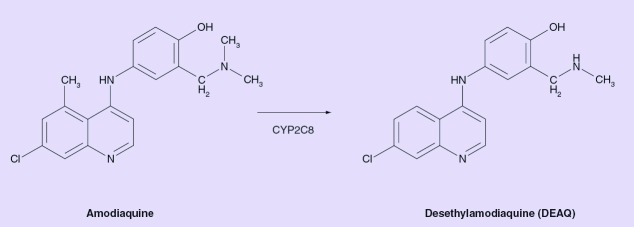
Amodiaquine and its main metabolite desethylamodiaquine. CYP: Cytochrome P450.

Malaria, AIDS and tuberculosis (TB) are the three top killing communicable diseases. Malaria alone claims at least one life every 30 s. The overall burden of malaria can reach 2 million casualties/year, mostly among children aged under 5 years and pregnant women in sub-Saharan Africa [1,2]. The human malaria agents are parasites belonging to the genus *Plasmodia*. The clinically most important among them are *P. vivax* and *P. falciparum*, the latter being responsible for the mortality associated with the disease.

Even after decades of research, the development of a vaccine with significant impact in the endemic populations is still many years away [3]. This leaves drug therapy as the main tool for the global treatment and control of the disease, presently in the successful shape of the highly effective combination of artemisinine (ART) derivatives with longer half-life partners [4].

Amodiaquine (AQ), a 4-aminoquinoline, is one of the most important drugs for malaria control in the African continent, being the basis of the first-line treatment in 15 countries as an ART combination therapy (ACT) drug partner (AQ-artesunate).

AQ is a very specific substrate of cytochrome P450 (CYP)2C8 [5], to the extent of constituting a potential *in vivo* probe drug for this enzyme [6]. CYP2C8 has been also demonstrated *in vitro* to metabolize the presently less relevant antimalarial drugs chloroquine (CQ) and dapsone [7,8].

Compared with their importance, the body of knowledge concerning the enzymes involved in the metabolism of these antimalarials is disproportionately small [9]. Accordingly, *in vivo* data regarding the relation of the efficacy of these drugs with pharmacogenetics is generally not available.

The objective of this review is to contribute to filling this gap by inferring from the available data the relevance – or lack of it – of the *CYP2C8* gene and its polymorphisms in the presently applied chemotherapies, with particular focus on AQ. This will be described in the context of the current knowledge concerning parasite multidrug resistance mechanisms and the specific scenario of malaria as a major infectious disease.

Unless otherwise stated, this review focuses on *P. falciparum* malaria.

## CYP2C8 & its coding gene

The *CYP2C* genes are located in a cluster at chromosome 10q24, organized as Cent–*CYP2C18*–*CYP2C19*–*CYP2C9*–*CYP2C8*–Tel [10]. The *CYP2C8* gene spans approximately 30 kb, including nine exons (Figure 1) [11]. This open reading frame codes for a 490 amino acid protein, which quantitatively represents approximately 7% of the total liver CYP enzymes and approximately 30% of the hepatic CYP2C subfamily content. Among the major members of the CYP2C subfamily (2C8, 2C9 and 2C19), CYP2C8 is the most divergent in terms of protein sequence: while CYP2C9 and CYP2C19 share over 90% of homology, CYP2C8 is only 75% similar to each one of these enzymes. Notably, the CYP2C8 active site has recently been shown to be significantly different from the other members, in size (1438 Å^3^) and shape [12]. The volume of the active pocket allows the accommodation of relatively large molecules, probably contributing for the differences in ranges of substrates as compared with 2C9 and 2C19 [13].

*CYP2C8* transcription activity has been identified in extrahepatic tissues, including the kidneys, adrenal gland, uterus, brain [11,14], mammary glands, ovaries, duodenum [11], small intestine, lung, prostate, testis [14] and the heart [15]. The actual CYP2C8 protein has been immunologicaly identified in the kidneys, gut (small and large intestine), salivary glands, adrenal cortical cells and tonsils [16].

Several nonhomologous single nucleotide polymorphisms (SNPs) have been identified in the *CYP2C8* open reading frame, giving rise to at least ten formally distinct alleles [101]. Besides the alleles found in the original communications on *CYP2C8* biodiversity (*2,*3,*4,*5) [17,18], the remaining were discovered in surveys conducted in Japan (*6–*10) [19–21]. *CYP2C8* null homozygous have not been described, pointing to the probable physiological importance of this gene.

CYP2C8*2 and *3 are the most frequent alleles found worldwide. The former is characteristic of populations of African origin (Table 1), while *3 is essentially present among Caucasians. Orientals seem to have very high prevalence of wild-type alleles, although more extensive studies are warranted (e.g., in the large populations of China and India). Both alleles code for enzymes with decreased performance: while the CYP2C8*2 allele was observed to cause a twofold increase on the K_M_ for paclitaxel transformation [17]; *3 was documented to be associated with an arachidonic acid hydroxylation turnover of only 10% as compared with the wild-type allele (*1) [17].

In Figure 1, the structure of the *CYP2C8* gene is presented, as well as a compilation of SNPs published or readily accessible in several internet-accessible databases. In Table 1, the available data on the frequencies of the main studied alleles (*2, *3 and *4) in malaria endemic regions is presented.

## Parasite intraerythrocytic life cycle

Malaria infection starts with the injection of malaria sporozoites during the blood meal of an infected female mosquito (*Anopheles* spp.). In 30 min these forms will invade the liver. In 5–15 days, liver schizontes will mature, each one giving rise to tens of thousands of merozoites. These are released in the blood stream, where they promptly invade red blood cells, initiating the intraerythrocytic cycle. Asexual multiplication progresses in this cycle, with invading merozoites growing into trophozoites that will mature towards multinuclear intraerythrocytic schizonts. Each of these will provide 8–24 new merozoites, which are released at the burst of the erythrocytes. These will attack new red blood cells, re-initiating the typical (*P. falciparum*, and *P. vivax*) 48 hour intraerythrocyte cycle. During this process some merozoites will develop into gametocytes, sexual forms that, if ingested by the mosquito during the blood meal, will progress into fertilization and production of new sporozoites, ready for re-invading the human host and completing the overall cycle. In the case of *P. vivax*, the parasites can stay dormant in the liver for years before developing blood-stage infections.

All drugs herein discussed act in the intraerythrocyte cycle.

## Concept of artemisinine combination therapy

ACT is presently being adopted by most malaria-endemic countries for the control of the disease. The combination has its basis on the observation that ART represents the most powerful class of antimalarials in use in terms of parasite reduction ratio (PRR) [22], that is, the ratio of the parasitemia before the initiation of the treatment with the value registered 48 h later (one *P. falciparum* intraerythrocytic cycle). This has been shown to be approximately 10^4^ (reduction to 0.01% of the initial parasite load), which is significantly different from, for example, the 10^2^–10^3^ value characteristic of mefloquine. Although rapid killers, ART have short half-lives (0.5–2 h), implicating a characteristically long monotherapy treatment course for any coadministrated antimalarial.

In ACT, ART is combined with a long half-life antimalarial expected to have a different mechanism of action (e.g., AQ, of importance in this review). The basic rationale (Figure 2) is [22]:

• ART acts very fast, significantly reducing the parasite load in the first hours of treatment;
• The slowly eliminated partner drug (e.g., a quinoline antimalarial) is left to face a much decreased number of parasites;
• As the mechanisms of action of ART are expected to be different from the partner drug, the latter also acts over a particularly sensitive surviving population of parasites potentially selected by ART;
• The effect is further enhanced by the frequent pharmacodynamic synergism between its components (e.g., artesunate and mefloquine), which constitutes a frequent further advantage of ACT.

ACT is an actual combination for a relatively short period of time due to the short half-life of ART. Some hours after the administration of ACT, the partner drug is essentially alone in circulation, that is, ACT becomes a monotherapy. In regions of low malaria transmission (e.g., most of South East Asia) the risk of re-infection is low, so the long half-life partner drug will not interact with new parasites until it is totally eliminated. The situation in high transmission zones is different – a cured patient can be re-infected in just some days, at a point when the plasma concentrations of the long half-life partner drug are at subtherapeutic levels. The new incoming parasites will be exposed to low drug concentrations that prompt the selection of low-resistance ones (‘tolerant parasites’) [23]. Progressively, the population is expectedly stepwise selected to ever higher levels of tolerance until it reaches the previously established therapeutic window, generating clinical failure (*in vivo* resistance).

## Amodiaquine: a main partner in artemisinine combination therapy

AQ was synthesized for the first time over 40 years ago. The drug is, in general, clinically effective against chloroquine-resistant strains, although cross-resistance with this drug has been documented for at least 20 years [24]. AQ is a major combination therapy partner, being mainly employed as ACT with the artemisinine derivative artesunate, being a main first-line treatment in West African countries in particular [102]. AQ is usually given in combination with artesunate as oral doses of 10 mg/kg AQ and 4 mg/kg artesunate once daily for 3 days. The two drugs are presently supplied separately. A future convenient AQ-artesunate fixed combination (Coarsucam™) is being developed by Sanofi-Aventis in collaboration with Medicine for Malaria Venture [103] and is presently under efficacy trials [104]. Its expected low price – less than US$0.5 for a children treatment course – will probably command an increase of its use in African populations.

An alternative combination, AQ–sulfadoxine–pyrimethamine, has also been advocated, particularly for countries not ready to afford the rapid implementation of ACT [25]. This combination is currently being employed under official Malaria Control Programs in Papua New Guinea and Colombia.

## Pharmacokinetics of amodiaquine

Although the main target patient population is pediatric, AQ pharmacokinetics has mainly been studied in adults. This should be kept in mind when extrapolating the pharmacokinetic data to the clinical situation as the children may have higher or lower clearance/kg body weight, depending on their age and stage of maturation.

The plasma concentration time course is biphasic with a fast distribution phase and a slower elimination phase. After an intravenous 4-h infusion of 10 mg base/kg to ten adult patients with *P. falciparum* malaria, the half-life of the first disposition phase was 22 min (geometric mean; range 5–126 min) and the half-life of the terminal phase 10 h (geometric mean; range 3–33 h). Clearance was 6 l/h/kg (range 2–17) [22]. After an intravenous injection of 3 mg base/kg to six healthy volunteers, similar characteristics were observed but clearance was higher (geometric mean 13 l/h/kg; range 5–56) and the terminal half-life shorter (geometric mean 2 h, range 0.5–6 h) than in patients. These differences in observed clearance may be due to a reduced hepatic blood-flow or reduced enzyme activity in acute malaria. It is possible that there is another disposition phase of AQ with a longer half-life. This is indicated by the detection of AQ in urine for longer time than expected by Winstanley and colleagues [26]. The authors only present long-term urinary data for one subject, but in this subject 0.5 µg/ml AQ was observed 5 months after a 600 mg oral single dose.

AQ pharmacokinetics is independent of dose in the dose-range 200–600 mg administered as single doses to healthy volunteers. The drug appears to be eliminated mainly through metabolism with some contribution of renal excretion [26]. The main metabolite observed is the pharmacologicaly active desethylamodiaquine (DEAQ) (Figure 3), which is found in large amounts in the circulation. Two other metabolites, 2-hydroxy-DEAQ and *N*-bisdesenthyl-AQ, have also been found in the circulation in similar or higher concentrations than AQ [27,28].

After oral administration, the exposure of DEAQ is many-fold higher than the exposure of AQ. After a 600 mg oral dose of AQ to healthy volunteers, the DEAQ:AQ area under the plasma–time curve (AUC)_0–∞_ ratio was approximately 52 (the estimate is rough as both AUC_0–∞_ values included a large area extrapolated from the last data point). DEAQ accumulates in the red blood cells, reaching a red blood cells/plasma ratio of 3 [26]. Both AQ and DEAQ are highly (>90%) protein-bound [29]. DEAQ is assumed to be the main entity responsible for the therapeutic effect of AQ treatment. The disposition of DEAQ in healthy volunteers after a 10 mg/kg oral dose of AQ base has been observed to have three phases; two initial disposition phases with half-lives of 3–11 h and 23–50 h and a terminal phase with a half-life of 9–18 days in plasma and 12–32 days in whole blood [30].

*In vitro* metabolism studies with enzyme expression systems, human liver microsomes, enzyme activity correlation analysis and enzyme docking analysis, strongly indicate that CYP2C8 is the only hepatic CYP isoform involved in the formation of DEAQ [5], and the main hepatic enzyme responsible for AQ metabolism. DEAQ appears to be eliminated through renal excretion as well as further metabolized by other, probably extrahepatic, CYP enzymes, namely CYP1A1 [5] (I Cavaco, University of Algarve, Faro, Portugal. Unpublished data).

## *CYP2C8* polymorphisms probably influence amodiaquine metabolism

There is, as yet, no direct demonstration of the influence of the CYP2C8 inhibition or polymorphism on the pharmacokinetics, efficacy and safety of AQ in malaria settings. An expected importance of the polymorphism stems from the following observations:

• CYP2C8 is essentially the sole hepatic CYP enzyme catalyzing the formation *in vitro* of DEAQ [5];
• Significant variability in the pharmacokinetics of AQ has been documented [26,31];
• The main studied *CYP2C8* polymorphisms are associated with significant alterations in *in vitro* enzyme activity [17,18,32].

The relation between these alleles and *in vivo* pharmacokinetics (PK) parameters is less clear, partly because of the fact that the tested drugs are also metabolized by other CYPs. In fact, although some reports did find significant difference in the PK of the drugs under study [33–35], others did not observe it, albeit with other CYP2C8 substrates [36–38]. It is important to emphasize that AQ is actually one of the best candidates as an *in vivo* CYP2C8 probe substrate [6].

From this scenario, it is presently only possible to predict the importance of CYP2C8 for the efficacy of AQ. For this purpose it is important to also take into account some characteristics of the variable response of the pathogen to AQ and DEAQ.

## *P. falciparum* amodiaquine/ desethylamodiaquine resistance

Malaria therapy with AQ is in most cases effective against CQ resistance *P. falciparum*. Nevertheless, events of the parasite being simultaneously refractory *in vivo* to both drugs are not rare, pointing for a certain degree of cross-resistance [24].

*In vivo P. falciparum* resistance to AQ regimens seems to be associated with mutations in the parasite *P. falciparum* CQ resistance transporter (*Pfcrt*) gene [39], coherent with the occasional observation of CQ/AQ clinical cross-resistance.

*Pfcrt* codes for a transmembrane protein located in the parasite food vacuole, the central organelle in the metabolism of hemoglobin, an essential requirement for the progression of the parasite cycle. During the metabolism of hemoglobin the toxic heme is liberated from its molecular scaffold. CQ (and possibly AQ or DEAQ) binds heme, disturbing its inactivation by the parasite [40]. *Pfcrt* SNPs, in particular a K76T variation, render the parasite resistant to the drug, probably through the efflux of CQ from the organelle. CQ-resistance is reversible *in vitro* by the action of the calcium blocker verapamil (VP), known to interfere specifically with K76T harboring PfCRT proteins. Interestingly, although DEAQ sensitivity is reversible with VP, AQ response is not significantly affected [41]. This data points for *pfcrt* K76T to be associated mainly with DEAQ response, partially explaining previously observed *in vivo* CQ/AQ cross-resistance *in vivo*, which should maybe be better interpreted as CQ/DEAQ. Furthermore, due to the aforementioned ‘tail effect’, the slowly eliminated DEAQ is predicted to be the weak link promoting resistance in the AQ–ART combination.

## Can *CYP2C8* polymorphisms influence amodiaquine regimens efficacy & safety?

The effect of *CYP2C8* polymorphism on DEAQ AUC is dependent on the contribution of this pathway to AQ total clearance. *In vitro*, CYP2C8 was found to be the main hepatic CYP involved in the metabolism of this drug. However, some contribution of the extrahepatic enzymes CYP1A1 and CYP1B1 was also observed [5]. No *in vivo* data on the contribution of different elimination pathways has been documented. If the extrahepatic pathways are considered likely to be of minor importance for total clearance, DEAQ AUC will not be significantly affected by *CYP2C8* polymorphism. The DEAQ formation will be slowly giving rise to some decrease in maximum plasma concentration (C_max_), with the AUC not expected to be affected.

AQ is a more active antimalarial then DEAQ, with half-maximal inhibitory concentration (IC_50_) values 2–8-fold lower [27,42,43]. The little information available shows that the C_max_ of AQ is relatively low – approximately 30 nM (600 mg dose) – [44], falling anyway inside the *in vitro* IC_50_ values of a significant fraction of field analyzed AQ sensitive parasites [41,45–49]. This suggests that AQ might give a small but non-negligible contribution for the success of the therapy, particularly among a predicted *CYP2C8* ‘low-metabolizer’ genotype carrier associated with an increased drug exposure for the parasite. The positive effect of an increased AQ exposure might not be evident in these early days of high efficacy ACT, as observed in a small trial of 20 patients in Papua New Guinea [50]. In this study, all patients were determined as *CYP2C8* wild type and were successfully treated, the authors concluding that “the AQ regimen seems pharmacokinetically adequate in the absence of *CYP2C8* polymorphism” [50]. Nevertheless, with the potential development of *P. falciparum* resistance to DEAQ, the benefit of a high AQ exposure might increase.

This contribution is probably more important through another venue: *in vitro* experiments have consistently shown that AQ is capable of marked synergism with artemsinine derivatives [51,52] and, most notably, with its own DEAQ metabolite [43]. Again, the presence of *CYP2C8* alleles predisposing for a higher AQ exposure will be expected to prolong the synergistic influence over DEAQ. Results *in vitro* show that AQ is able to significantly enhance the action of its metabolite towards extraordinary low AQ:DEAQ ratios, beyond 1:100,000. This observation implicates that although AQ serum levels generally drops below the present level of detection (approximately 10 nM) 8 h after administration, very low concentrations – down to the picomolar range – may significantly aid DEAQ antiparasitic action for several days. Again, low-metabolizer patients are potentially associated with an enhanced performance of the AQ chemotherapy, possibly even in areas where DEAQ resistance might be rising, as this synergistic effect seemed to be independent at least of the *pfcrt* K76T status of the tested parasites [43].

In the 1980s, reports documented fatal adverse drug reactions among travelers under AQ-based prophylaxis (incidence 1:2000), mainly through agranulocytosis and hepatic toxicity [53]. This led to a WHO recommendation towards the withdrawal of AQ from malaria control programs, a decision later considered ‘more prudent than practical’ [54].

*In vitro* studies have shown that when compared with DEAQ, AQ is more frequently transformed to the highly reactive quinoneimine species proposed as responsible for adverse events associated with AQ profilaxis [55–57]. Hence, being a low-metabolizer potentially elevates the risk of AQ associated serious side effects [5].

Treatment with AQ has not been documented to be associated with severe adverse effects, showing to be not more toxic then CQ [54,58]. However, more time and further pharmacovigilance efforts are needed for reliable conclusions to be drawn [59], in particular in a scenario of possible increase in AQ doses following a significant decrease in parasite susceptibility to this chemotherapy. These can be aided by the identification of carriers of *CYP2C8* alleles predicted to be associated with AQ low metabolism. In the African continent, the analysis of the *2 allele would be sufficient to identify most of these. *2/*2 carrying patients could be selected for enhanced surveillance.

## Interactions

*CYP2C8* has been shown to be readily inducible *in vitro* via nuclear receptors pregnane X receptor (PXR), constitutive androstane receptor (CAR) and glucocorticoid receptor (GR) activators [60]. Recently, it has been demonstrated that artemisinine, artemether and artether derivatives are potent PXR/CAR agonists, leading to the induction of the CYP2B6, CYP3A4 and MDR1 in primary hepatocytes [61], as well as to increased CYP2B10 and CYP2A5 enzyme activities *in vivo* in a rodent model [62]. Although the authors did not test the induction potency of artesunate, neither the induction of CYP2C8, the possibility remains that when used in ACT, AQ might be metabolized at a higher rate during ACT due to artesunate-based activation of PXR and/or CAR [61]. Furthermore, it is also possible that other commonly co-administered PXR activators, such as the antituberculosis agent rifampicin and some of the HIV drugs, may further induce the CYP2C8 expression. Such interactions are likely to reduce AQ exposure. In this scenario, the relative positive impact of low-metabolizer status in the efficacy of AQ might still be present, as a less active enzyme will be induced as compared with an induced fast-metabolizer.

The importance of CYP2C8 for drug metabolism has not been acknowledged until recently; hence few drugs are known to markedly inhibit CYP2C8 *in vivo*. A number of drugs have been identified as potent CYP2C8 inhibitors *in vitro*, with predicted risks of drug–drug interactions with substrates of this enzyme having been calculated (inhibitor concentration/inhibition constant values) [63]. Some CYP2C8 inhibitors are under therapeutic use in areas where AQ is also being administered for malaria treatment, including ritonavir (anti-HIV drug), ketoconazole (antifungal) and the antibacterial thrimethoprim.

AQ low-metabolizers can possibly be at additional risk due to their lower enzyme activity during cotreatment with CYP2C8 inhibitors.

## Chloroquine – the old mainstay

Due to the development of parasite resistance, CQ is presently being officially phased out in most endemic countries. It is still part of the national malaria control strategy in several countries, for example, Congo Brazzaville and Togo [102]. In other regions, although other drugs have been adopted, their implementation will still take significant time extending the use of this drug. CQ is still a main drug of choice at a global level for the treatment of *P. vivax* malaria.

## Chloroquine pharmacokinetics

CQ is well absorbed after oral administration and has a bioavailability of approximately 80% [64]. CQ is eliminated through metabolism, as well as renal excretion, the latter pathway accounting for approximately 50% of total drug clearance [65]. The main metabolite of CQ is *N*-desethylchloroquine [64]. Other metabolites, such as bisdesenthyl chloroquine and 7-chloro-4-aminoquinolone, are also formed. Bisdesenthyl chloroquine is present in concentrations approximately 40% of those of CQ. The metabolite is pharmacologically active and has been proposed to be further metabolized by the enzyme also catalyzing its formation [66]. CQ exhibits a very long terminal half-life (up to 58 days). The length of the half-life is mainly caused by its very large volume of distribution (up to 1000 l/kg), as the clearance of the drug is relatively rapid (up to 1 l/h/kg). CQ is metabolized *in vitro* by CYP2C8 and CYP3A4, with a non-negligible contribution of CYP2D6, particularly at drug concentrations below 10 µM. [7]. The quantitative contributions of these enzymes *in vivo* are not clear.

Due to the large contribution of renal excretion and the multiple enzymes involved, at least *in vitro*, the effect of CYP2C8 polymorphism is not expected to be marked in patients with a normal renal function. It is to note that patients with malaria have been observed to have a minor impairment of renal function [67]. However, the impairment may be too small to significantly affect CQ excretion.

## Dapsone

Dapsone is, with chlorproguanil, a part of the antifolate Lapdap™ combination, developed as a future replacement of sulfadoxine–pyrimethamine, as it is active against parasites resistant to this drug [68]. Resistance is expected to develop slower than with sulfadoxine–pyrimethamine, as both dapsone and chlorproguanil have significantly shorter half-lives, hence avoiding the ‘tail effect’ [69]. Unfortunately, the mechanism of resistance to these drugs is probably similar to the associated sulfadoxine–pyrimethamine (SNPs in *P. falciparum* dihydrofolate reductase and dihydropteroate synthase coding genes), well known to be particularly fast to rise [70].

Dapsone is reversibly metabolized to monoacetyldapsone, but the major pathway appears to be the formation of dapsone hydroxylamine, which has been associated with toxicity. The latter is CYP-catalyzed [71]. *In vitro* studies performed at clinically relevant concentrations of dapsone (4 µM) have shown that the drug is mainly metabolized by CYP2C9, with an expected minor CYP2C8 contribution [8]. This data, although not yet confirmed *in vivo*, points for a minor contribution of genetic *CYP2C8* variability for the pharmacokinetics, efficacy and safety of the drug.

## Expert commentary

Antimalarials are administered in high numbers in the developing world, in many settings as a default for most fever events without confirmation of the presence of the parasite. In a time when large changes in the national drug policies are underway, a more detailed knowledge on host factors influencing the efficacy and safety of the ACT partner drugs should be mandatory. This includes the understanding of the pharmacogenetic characteristics of the target populations.

CYP2C8 appears to be the only hepatic CYP enzyme metabolizing AQ, suggesting that polymorphisms in this gene can be useful tools for predicting individual exposure to the drug. Unfortunately, the importance of its polymorphism in the individual exposure to AQ is still to be experimentally established. Furthermore, the bulk of data concerning the pharmacokinetics of AQ is almost two decades old, involving only small studies, mainly with adult subjects. New investigations adapted to the present clinical scenario of the use of AQ in Africa are as urgent as fundamental.

In case of its development, such molecular tools might be used as a convenient way for sampling populations under AQ-based first-line chemotherapies, determining the likelihood of a patient to come under higher-than-average exposure. This can be of importance in eventual changes in dosing, for example, due to increased *P. falciparum* DEAQ tolerance. This would also be valuable information for the future introduction of other drugs also metabolized by CYP2C8.

Nevertheless, it is to note that in this moment, when *P. falciparum* resistance to the newly employed drugs is still not maturated, the influence of CYP2C8 pharmacogenetics in the efficacy of AQ-based regimens is less likely to be important. However, the increased exposure to AQ in poor-metabolizers may predispose to adverse events.

Finally, the influence of *CYP2C8* polymorphism in the therapeutic efficacy of CQ and dapsone is not expected to be significant.

## Future perspective

*P. falciparum* has shown in the last decade to be a resilient pathogen, able to timely develop resistance to every applied antimalarial. In the long term, it is not unexpected that the same will happen with the presently used chemotherapies. In the specific case of AQ, a shrinking of the therapeutic window in the next years, particularly through falling efficacy of the long half-life DEAQ, might highlight the importance of interindividual differences in AQ exposure.

Future research will clarify the influence of *CYP2C8* polymorphisms in the modulation of AQ exposure and the associated clinical efficacy, and potential risk of adverse reactions. In the next 10 years, this might permit the development of *CYP2C8* allele analysis as one of a set of convenient molecular tools aiding pharmacovigilance programs of massive employed drugs in the developing world. In the case of Africa, where most of the AQ is presently being used, the main non-wild-type allele present is CYP2C8*2, leading to the notion that the sole analysis of the I269F SNP will cover most of the predicted low-metabolizer individuals. This simplified approach can facilitate the logistics of the future implementation of this type of tool, potentially important in the definition of groups with an expected different therapeutic window from the *1/*1 carrier majority. Adjustments of the drug dosage might be of interest for exploiting in the best way the pharmacokinetic characteristics of this minority group.

**Table 1.** Published prevalences of the main *CYP2C8* alleles in malaria-endemic regions.

Allele‡	Malaria-affected regions		Comparators^*^						
	Zanzibar (n = 16)	Ghana (n = 200)	Malaysia (n = 57)	Papua New Guinea (n = 305)	SE Asia (n = 20)		African–Americans	Western Europeans	Japanese
CYP2C8*2	0.139	0.168	0.035	0.000	0.000		0.180–0.150	0.004–0.016	0.000
CYP2C8*3	0.021	0.000	0.053	0.000	0.050		0.020–0.080	0.069–0.198	0.000–0.007
CYP2C8*4	0.006	0.000	0.000	0.000	0.000		0.000	0.045–0.075	0.000
LM	0.036	0.015	<0.005	0.000	0.000		0–0.019	0–0.067	0.000
Ref.	[72]	[73]	[74]	[50]	[75]		[17,76,77]	[78]	[19,21,79]

^*^Data concerning average values derived from studies in African–American, Japanese and Western Europeans are given as comparators. ^‡^Other rare alleles were essentially only found in Japanese: *5 = 0.003 [76] and 0.009 [19]; *6 = 0.003; *7 = 0.003; *8 = 0.003; *9 = 0.003; *10 = 0.003 [21]. CYP: Cytochrome P450; LM: Low-metabolizer, inferred from the frequency of subjects homozygous for the mutant alleles.

**Highlights**

• Cytochrome P450 (CYP)2C8 is essentially the sole CYP enzyme involved in *in vitro* amodiaquine (AQ) metabolism.
• The *CYP2C8* gene shows polymorphism. Null alleles are known, but are very rare. Over 95% of the mutant allele frequency is explained in most populations by the presence of three variants (*2, *3 and *4). These are expected to influence AQ exposure, although the present data indicates that the exposure to desethylamodiaquine (DEAQ) will be approximately the same. Studies formally confirming this hypothesis are still missing, not currently allowing a precise picture to be formed.
• The efficacy of AQ seems to be presently satisfactory in those homozygous for the CYP2C8*1 allele, although more data needs to be gathered, particularly concerning the AQ–artesunate combination. Clinical outcome is more certain to be influenced by parasite characteristics and other factors. However, variable CYP2C8 activity can become important in the future event of increased *P. falciparum* insensitivity to DEAQ: low-metabolizers will have more contribution of AQ in the elimination of DEAQ-tolerant parasites, particularly through the marked synergy of AQ with DEAQ and artesunate.
• On the other hand, AQ poor-metabolizers are probably at higher risk of adverse events related to this drug. This may prove to be even more important if the AQ dose is increased in order to circumvent DEAQ tolerance.
• CYP2C8 is probably of low relevance in the performance of chloroquine and dapsone.
